# Characterization of Glutathione Dithiophosphates as Long-Acting H_2_S Donors

**DOI:** 10.3390/ijms241311063

**Published:** 2023-07-04

**Authors:** Rezeda A. Ishkaeva, Nail N. Khaertdinov, Aleksey V. Yakovlev, Marina V. Esmeteva, Diana V. Salakhieva, Ilyas S. Nizamov, Guzel F. Sitdikova, Timur I. Abdullin

**Affiliations:** 1 Institute of Fundamental Medicine and Biology, Kazan (Volga Region) Federal University, 18 Kremlyovskaya St., 420008 Kazan, Russia; rezaahmadishina@kpfu.ru (R.A.I.); nail.khaertdinov@kpfu.ru (N.N.K.); aleksey.yakovlev@kpfu.ru (A.V.Y.); mavesmeteva@kpfu.ru (M.V.E.); divsalahieva@kpfu.ru (D.V.S.); timur.abdullin@kpfu.ru (T.I.A.); 2 Scientific and Educational Center of Pharmaceutics, Kazan (Volga Region) Federal University, 18 Kremlyovskaya St., 420008 Kazan, Russia; 3 Alexander Butlerov Institute of Chemistry, Kazan (Volga Region) Federal University, 420008 Kazan, Russia; ilyas.nizamov@kpfu.ru; 4 Arbuzov Institute of Organic and Physical Chemistry, FRC Kazan Scientific Center of RAS, 8 Arbuzov St., 420088 Kazan, Russia

**Keywords:** hydrogen sulfide, donors, glutathione, dithiophosphates, myoblasts, myocytes, reactive oxygen species, atrial myocardium, contraction, negative inotropic effect

## Abstract

Considering the important cytoprotective and signaling roles but relatively narrow therapeutic index of hydrogen sulfide (H_2_S), advanced H_2_S donors are required to achieve a therapeutic effect. In this study, we proposed glutathione dithiophosphates as new combination donors of H_2_S and glutathione. The kinetics of H_2_S formation in dithiophosphate solutions suggested a continuous H_2_S release by the donors, which was higher for the dithiophosphate of reduced glutathione than oxidized glutathione. The compounds, unlike NaHS, inhibited the proliferation of C2C12 myoblasts at submillimolar concentrations due to an efficient increase in intracellular H_2_S. The H_2_S donors more profoundly affected reactive oxygen species and reduced glutathione levels in C2C12 myocytes, in which these parameters were elevated compared to myoblasts. Oxidized glutathione dithiophosphate as well as control donors exerted antioxidant action toward myocytes, whereas the effect of reduced glutathione dithiophosphate at (sub-)micromolar concentrations was rather modulating. This dithiophosphate showed an enhanced negative inotropic effect mediated by H_2_S upon contraction of the atrial myocardium, furthermore, its activity was prolonged and reluctant for washing. These findings identify glutathione dithiophosphates as redox-modulating H_2_S donors with long-acting profile, which are of interest for further pharmacological investigation.

## 1. Introduction

As a gasotransmitter, H_2_S plays an important regulatory role in various physiological processes and functions in cardiovascular, nervous, gastrointestinal and other systems [1,2]. Some mechanisms of H_2_S action involve the regulation of membrane potential via interaction with ion channels and membrane receptors, gene expression through several pathways (PI3K/Akt, NF-κB, MAPK), cellular bioenergetics and proliferation, endocytotic and exocytotic processes [2,3,4]. H_2_S signaling is mediated by its redox reactions with cysteine residues in proteins [5] and formation of labile metabolites such as persulfides and (hydro-)polysulfides [6,7]. H_2_S has documented antioxidant and cytoprotective properties attributed to a direct reducing and radical-scavenging ability as well as the conversion of endogenic thiols to sulfane sulfur species with increased reactivity and lower susceptibility to irreversible oxidation [6,8].

Impaired H_2_S biosynthesis is implicated in different pathological conditions often associated with decreased expression and activity of H_2_S-synthesizing enzymes, namely cystathionine β-synthase (CBS) and cystathionine γ-lyase (CSE). Decreased H_2_S levels were reported upon hypertension [9], diabetes, neurodegenerative diseases [10] and myocardial ischemia-reperfusion injury (IRI) [11].

Blood plasma H_2_S levels were shown to be significantly lower in patients with coronary heart disease and heart failure in comparison with healthy individuals [12]. Both the plasma concentration of H_2_S and plasma capability for H_2_S production were reduced in streptozotocin-treated diabetic rats. Similarly, decreased H_2_S in the plasma of type 2 diabetic patients was reported [13].

The use of exogenic H_2_S in the form of donors was recognized as an important strategy for treating the aforementioned and other diseases. The state of the art in bioactive H_2_S donors is presented in recent reviews [1,14]. A series of proof-of-concept studies on the pharmacological effects of H_2_S in vitro and in vivo was performed using inorganic sulfide donors such as NaHS and Na_2_S [15,16,17,18,19,20,21,22]. These donors provide fast extracellular release of H_2_S and therefore have restricted therapeutic potential. Garlic-derived polysulfides were shown to mitigate IRI in mice and hyperglycemia-induced reactive oxygen species (ROS)-mediated apoptosis but were considered relatively toxic [23].

Particular efforts were focused on the development of advanced H_2_S donors generally in the form of pro-drugs or hybrid molecules with existing drugs, which are intensively studied in preclinical models, and some of them progressed into clinical trials [15]. In particular, ACS15 represents a diclofenac conjugate with H_2_S-releasing dithiol-thione moiety with enhanced multiple anti-inflammatory effects and decreased gastric toxicity [24].

Many H_2_S-releasing agents are reductant-sensitive so that abundant natural thiols such as cysteine-containing molecules can promote slow H_2_S generation [25]. A series of thiol-responsive H_2_S-releasing agents were designed on the base of perthiol [26], arylthioamide [27] and *N*-(benzoylthio) benzamide [28]. These donors were shown to protect myocardium upon IRI in mice [26] and exert beneficial effects in cardiovascular in vitro and in vivo models including a reduction in systolic blood pressure [27]. In spite of encouraging preclinical data, the therapeutic potential of such thiol-activated compounds can be restricted by deficient reduced glutathione (GSH) levels in cardiovascular diseases [29].

As a part of other approaches to the development of H_2_S pro-drugs, enzyme-sensitive agents (e.g., HP-101, Esterase-TCM-SA, NTR-H_2_S) [30] and pH-sensitive ones (e.g., JK-1~5) [31] were synthesized. In particular, the latter donors have been designed to preferably release H_2_S at low acidic conditions (pH < 6) revealed during IRI to potentiate their local protective effect [31]. While most newly proposed H_2_S donors are laboratory-synthesized ones, there are few commercially available agents for research purposes, e.g., morpholin-4-ium 4 methoxyphenyl(morpholino) phosphinodithioate (GYY4137) [32]. This donor features a slow release rate, requiring increased doses to achieve therapeutic H_2_S levels, and thus poses increased risks of adverse effects [16,33,34].

There is still a need for improved H_2_S donors with greater specificity and safety profile. Considering physiologically relevant redox conversions between H_2_S and thiol-based molecules and their role in H_2_S signaling, a promising strategy to modulate the activity of H_2_S donors could rely on their combination with glutathione. In addition to H_2_S, exogenic glutathione and its derivatives have proven cytoprotective, antidegenerative, antiviral and other beneficial activities [35,36,37,38].

We recently synthesized glutathione dithiophosphates, which are bioactive amphiphilic ammonium salts of the tripeptide and dithiophosphoric acid capable of generating H_2_S, glutathione and products of their redox exchange [39]. Their properties as a H_2_S donor have not been elucidated to date. This primary study is focused on the characterization of the H_2_S-releasing profile of the glutathione dithiophosphates as well as their effects on muscle cell responses and myocardium contraction. The results suggest glutathione dithiophosphates as a new type of long-acting H_2_S donors, which are of considerable interest for further pharmacological investigation.

## 2. Results

### 2.1. Cytotoxicity of Glutathione Dithiophosphates

Glutathione dithiophosphates are salt compounds obtained as a result of the ionic interaction between the protonated NH_2_ group of Glu residue of glutathione and the ionized S-H group of O,O-dimenthyl derivative of dithiophosphoric acid (DTP). The dithiophosphates of reduced glutathione and oxidized glutathione (GSSG) were designated as GSH–DTP and GSSG–(DTP)_2_, respectively (for their structure, see Section 4.1. of Materials and Methods). Previously, the compounds were found to undergo gradual dissociation in aqueous solution accompanied by the hydrolysis of free DTP components to phosphoric acid, menthol and H_2_S [39].

**Figure 1:**
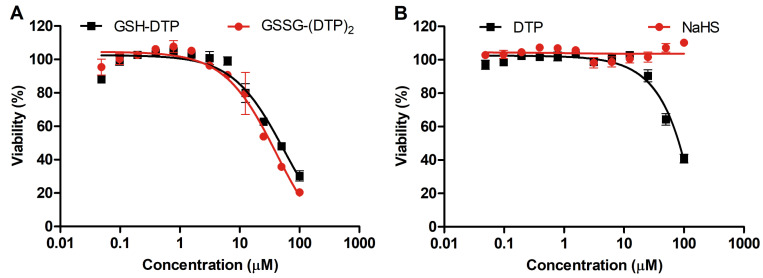
Effect of (**A**) glutathione dithiophosphates and (**B**) DTP and NaHS on viability of C2C12 myoblasts (MTT assay, 72 h).

According to the MTT assay, GSH–DTP and GSSG–(DTP)_2_ at submillimolar concentrations inhibited the proliferation of C2C12 murine myoblast cells with IC_50_ values of 56.5 ± 1.3 μM and 41.0 ± 1.2 μM, respectively (Figure 1). The control agents showed lower (DTP) and no activity (NaHS) under the same conditions. The effect of dithiophosphates on cell viability was attributed to the release of relatively cytotoxic H_2_S. The increased activity of GSH–DTP and GSSG–(DTP)_2_ could be associated with their H_2_S donation ability as well as the cellular availability of the unhydrolyzed compounds.

### 2.2. H_2_S-Releasing Profile

**Figure 2:**
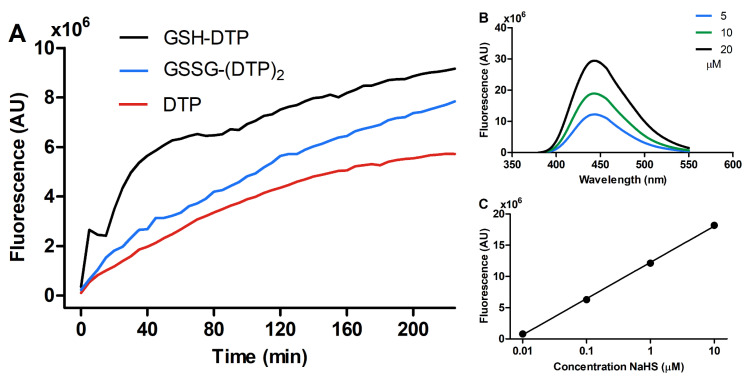
(**A**) Kinetics of H_2_S release upon hydrolysis of glutathione dithiophosphates (1 mM) in phosphate-buffered saline (PBS, pH = 7.4) according to fluorescence of AzMC (10 μM). Dependence of AzMC signal on (**B**) concentration of the probe (1 mM NaHS) and (**C**) concentration of NaHS (10 μM AzMC). For (**A**), the measurements were taken every 5 min.

The kinetics of H_2_S release by the glutathione dithiophosphates was studied using a 7-azido-4-methylcoumarin (AzMC) probe. The concentration of GSSG–(DTP)_2_ was normalized by one GSH/DTP moiety for a relevant comparison with GSH–DTP and DTP. The compounds were found to sustainably generate H_2_S with the following efficacy: DTP < GSSG–(DTP)_2_ < GSH–DTP (Figure 2A). GSH–DTP exhibited complex kinetics with a fast stepwise reaction during the first hour, which slowed down smoothly afterwards. GSSG–(DTP)_2_ and DTP provided smoother release profiles, where the reaction of DTP decayed more rapidly.

Unlike the synthesized compounds, NaHS, an immediate H_2_S donor, did not show a time-dependent increase in AzMC fluorescence. The relationships of this signal and the concentration of the probe and NaHS (Figure 2B,C) suggested that H_2_S content released by 1 mM glutathione dithiophosphates for 2 hrs was around 1 μM (given that ca. 11–13% of NaHS equivalents are converted into H_2_S [4]). These data show that GSH–DTP and GSSG–(DTP)_2_ are long-acting H_2_S donors with somewhat different release profiles.

### 2.3. Effect on Cellular H_2_S

**Figure 3:**
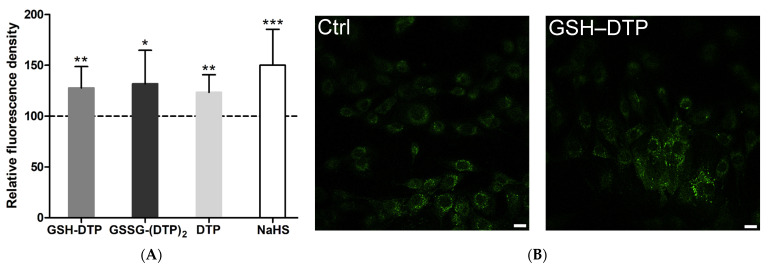
Effect of glutathione dithiophosphates, DTP and NaHS on H_2_S level in C2C12 myoblasts according to AzMC fluorescence. (**A**) Relative cell fluorescence intensity vs. control value of untreated cells (100%). (**B**) Representative LSCM images of control and GSH–DTP-treated cells. The pre-stained cells were exposed to dithiophosphates (10 μM) and NaHS (1 mM) for 20 min. Mean ± SD are shown (*n* = 12, * *p* < 0.05, ** *p* < 0.01, *** *p* < 0.001 vs. control). The scale bar is 10 μm.

Change in H_2_S content in C2C12 myoblasts exposed to the glutathione dithiophosphates was assessed at a pre-optimized concentration of 10 μM and exposure time of 20 min. Figure 3 shows the relative fluorescence intensities of the treated cells and representative laser scanning confocal microscopy (LSCM) images; other images are provided in Figure S1 (Supplementary Material). The results demonstrate that the synthesized donors are capable of a moderate increase in cellular H_2_S in a similar manner by 1.2–1.3 times. Under the same conditions, NaHS significantly elevated the H_2_S level only at an excessive concentration, i.e., 1 mM (by a factor of ca. 1.5) (Figure 3). Although these data do not allow us to compare the studied compounds, they suggest that the glutathione dithiophosphates are cellular H_2_S donors, which act at a lower concentration than NaHS.

### 2.4. Effect on Cellular GSH and ROS

**Figure 4:**
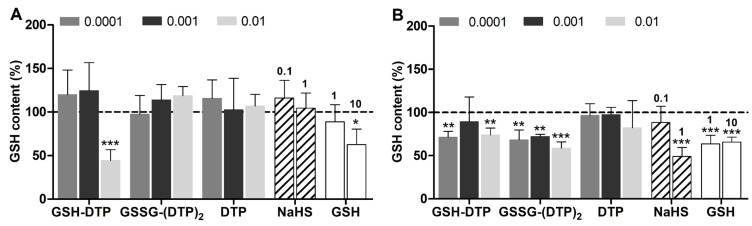
Relative GSH content in (**A**) C2C12 myoblasts and (**B**) C2C12 myocytes after 1 h exposure to H_2_S donors and GSH in HBSS according to MCB microplate assay [40] (vs. control values = 100%). Concentrations (mM) are shown in the legend (dithiophosphates) and above the diagram (NaHS, GSH). Mean ± SD are shown (*n* = 6, * *p* < 0.05, ** *p* < 0.01, *** *p* < 0.001).

**Figure 5:**
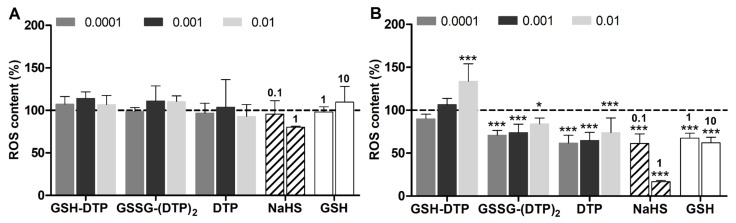
Relative ROS content in (**A**) C2C12 myoblasts and (**B**) C2C12 myocytes after 1 h exposure to H_2_S donors and GSH in HBSS according to DCFDA fluorescence (vs. control values = 100%). Concentrations (mM) are shown in the legend (dithiophosphates) and above the diagram (NaHS, GSH). Mean ± SD are shown (*n* = 4, * *p* < 0.05, *** *p* < 0.001).

Considering the existing relationships between cellular H_2_S and redox state, the effect of glutathione dithiophosphates on GSH and ROS content in C2C12 cells was assessed before and after cell differentiation. According to monochlorobimane (MCB) and DCFDA fluorecence, myocytes had higher GSH and ROS levels than myoblasts by ca. 1.2 and 5 times, respectively (Figure S2), reflecting the alteration of cell redox state after differentiation. Short-term cell exposure to the compared donors in an antioxidant-free solution (HBSS) resulted in the noticeable modulation of both GSH and ROS levels, i.e., their increase or decrease depending on conditions. In myoblasts, only GSH–DTP at an upper concentration of 10 μM caused a profound decrease in GSH content comparable to that observed for an excess of added GSH (10 mM) (Figure 4A). Such an effect can be attributed to the unbalanced increase in cellular GSH leading to reductive stress and decreased cell viability, although without noticeable ROS overproduction (Figure 5A).

In myocytes, both GSH–DTP and GSSG–(DTP)_2_ decreased the GSH level similarly in the concentration range studied (0.1–10 μM), whereas among the control agents, only 1 mM NaHS caused a comparable effect (Figure 4B). NaHS, DTP and GSSG–(DTP)_2_ showed inhibitory activity against overproduced ROS in myocytes, whereas GSH–DTP featured a modulating concentration-dependent effect from low antioxidant (0.1 μM) to noticeable prooxidant (10 μM) (Figure 5B).

Together, these data indicate that the glutathione dithiophosphates exhibit variable in vitro effects on GSH and ROS, which can be both anti- and prooxidant depending on the initial redox form and concentration of the compounds as well as the cell state.

### 2.5. Effect on Contractile Activity of Myocardium Stripes

**Figure 6:**
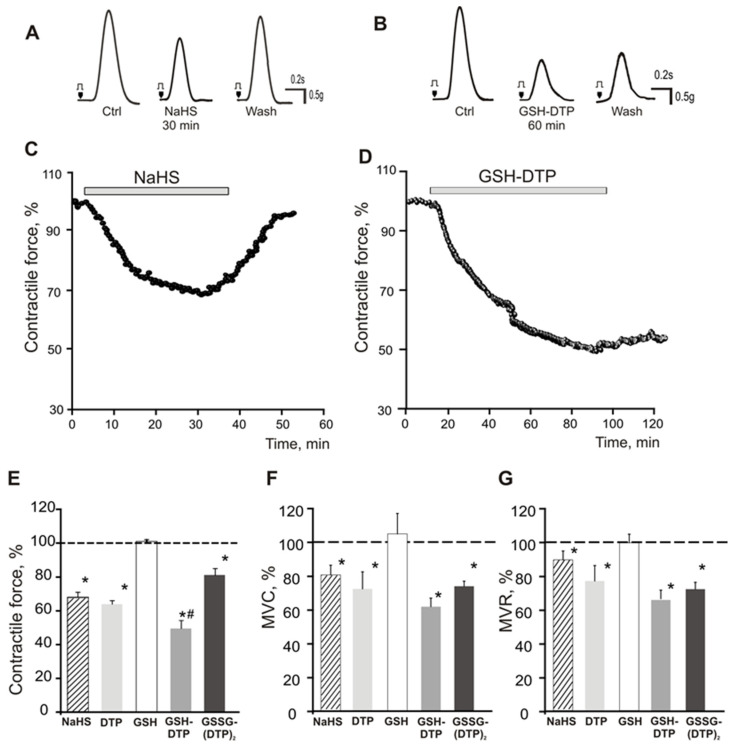
Effects of H_2_S donors on contractile force of atrial myocardium stripes. (**A**,**B**) Original traces of contraction curves of myocardium stripes in control (Ctrl), after treatment with H_2_S donors (NaHS or GSH–DTP, 200 µM) and washout (Wash). The time course of negative inotropic action of (**C**) NaHS and (**D**) GSH–DTP. (**E**–**G**) Maximum effects of NaHS (30 min), DTP (40 min), GSH (30 min), GSH–DTP (60 min) and GSSG–(DTP)_2_ (30 min) on (**E**) contractile force, (**F**) maximum velocity of contraction (MVC) and (**G**) maximum velocity of relaxation (MVR) vs. control (100%, dotted line). * *p* < 0.05 vs. control, # *p* < 0.05 vs. NaHS.

Contractile activity of isolated rat right atrial myocardium stripes exposed to the compounds was studied as established earlier [41,42]. The application of NaHS at a pre-optimized concentration of 200 μM induced a rapid H_2_S-mediated decrease in atria contraction (Figure 6A,C). The negative inotropic effect of NaHS was observed from the first minutes of application, while the contractile force decreased to an amplitude as follows (hereinafter, relative to initial values) 84 ± 2% (10 min), 73 ± 2% (20 min) and 68 ± 3% (30 min, *n* = 6; *p* < 0.05), which corresponded to the maximum effect maintained for up to 8 min (Figure 6C,E). Afterwards, the contraction amplitude started to recover due to H_2_S evaporation [4,43], and after washout, it was as high as 97 ± 4% by 20–30 min (*p* > 0.05, Figure 6C).

The treatment with 200 μM GSH did not significantly change the contractile force (101 ± 1%, *n* = 8) up to 60 min (Figure S3), whereas GSH–DTP and GSSG–(DTP)_2_ as well as DTP at the same concentration showed a profound negative inotropic effect attributed to H_2_S release.

DTP and GSSG–(DTP)_2_ decreased the contractile force relatively more slowly. In the case of DTP, the amplitudes were 90 ± 4% (10 min), 74 ± 2% (20 min) and 66 ± 2% (30 min) with a maximum effect of 64 ± 3% (*n* = 6, *p* < 0.05). The washout restored the amplitude to 74 ± 4% (*n* = 6, *p* < 0.05, Figure 6E). The corresponding values for GSSG–(DTP)_2_ were 92 ± 3% (10 min), 84 ± 4% (20 min) and finally 70 ± 6% (30 min). The washout restored the amplitude to 90 ± 6% (*n* = 9, *p* < 0.05; Figure 6E).

In the presence of GSH–DTP, the contractile force decreased to 81 ± 5% (10 min), 70 ± 6% (20 min), 63 ± 7% (30 min) and 53 ± 9% (60 min), whereas the amplitude was slightly recovered during washout (and at least 25 min afterward) to 57 ± 12% (*n* = 6, *p* < 0.05, Figure 6B,D). Moreover, all the DTP derivatives inhibited the maximum velocity of contraction (MVC) and relaxation (MVR) in a similar manner to NaHS, but to a somewhat higher extent (Figure 6F,G).

## 3. Discussion

This study identifies GSH–DTP and GSSG–(DTP)_2_ as effective H_2_S donors, which act at cellular and tissue levels. The compounds generate H_2_S in a sustained manner unlike fast-releasing salts such as NaHS/Na_2_S [44]. As was recently shown, they give rise to natural products in aqueous solutions [39] and expectedly have a decreased risk of adverse effects compared to xenobiotic donors. H_2_S is produced by the glutathione dithiophosphates upon gradual dissociation and hydrolysis of the DTP component, which in the case of free DTP occurs immediately. However, the current results suggest the continuous release of H_2_S in all dithiophosphate solutions (Figure 2A), indicating the formation of a DTP-derived sulfur-containing intermediate, which is gradually converted into H_2_S. The glutathione component of the dithiophosphates, although decreasing DTP hydrolysis [39], profoundly promoted H_2_S production by GSH–DTP (Figure 2A) presumably via a redox reaction of GSH with this intermediate. The latter reaction could explain a partial oxidation of the glutathione pool in GSH–DTP, whereas the interaction with generated H_2_S should underlie a partial reduction in this pool in GSSG–(DTP)_2_ according to previous data on redox conversions of the dithiophosphates [39]. Both reactions were accompanied by the formation of GSH persulfide (GSSH) (unpublished data), which is also in accordance with reported data on the interaction of glutathione with H_2_S and its derivatives [45,46,47].

Importantly, the glutathione dithiophosphates are envisaged as combination donors of H_2_S and GSH, both considered cytoprotective molecules. In addition to the therapeutic effects of H_2_S reviewed in the Section 1 [15,16,25,48,49], the administration of GSH per se or as prodrugs and derivatives was shown to provide beneficial effects upon different traumatic, degenerative and viral diseases [35,36,37,38].

To the best of our knowledge, there is a lack of reports on the joint therapeutic potential of H_2_S and glutathione [50]. We hypothesized that their effects can complement each other, e.g., via the generation of GSSH as a key mediator of H_2_S bioactivities, which reversibly traps and liberates H_2_S, reaching a relatively high concentration in the brain (>100 μM) and the myocardium (ca. 50 μM) [51]. Compared to GSH, GSSH possesses higher nucleophilic properties, making it a better antioxidant, but which may exhibit electrophilic behavior and act as a prooxidant [6]. In addition to providing GSH and H_2_S molecules involved in biorelevant interactions, the glutathione dithiophosphates have increased lipophilicity contributed by the menthyl groups of DTP and shielding the amino group of Glu residue upon salt formation. Menthol was selected to design the dithiophosphates as a pharmaceutically relevant but low-active modifier compared to other constituents. The highly hydrophilic nature of GSH restricts its passive diffusion across the cell plasma membrane, commonly requiring modification of the tripeptide or its combination with a carrier to improve cellular uptake [38].

The glutathione dithiophosphates are expected to provide increased effective concentrations of H_2_S, e.g., via mechanisms associated with cellular availability and sustained release of H_2_S. This is supported by the increased cell growth inhibitory activity of GSH–DTP and GSSG–(DTP)_2_ and, to a lesser extent, DTP compared to NaHS (Figure 1) and GSH. Presumably, the activity is determined by released H_2_S which exhibits cytotoxic and pro-apoptotic activities in increased doses due to the inhibition of complex IV of ETC and ATP production [52]. Furthermore, although at relatively low doses, both H_2_S [5] and glutathione [35] were shown to exert cyto- and tissue-protective properties, their excessive amounts may cause redox imbalance finally leading to oxidative stress [53,54].

According to AzMC fluorescence (Figure 3), the dithiophosphates were capable of a moderate increase in cellular H_2_S. The effect of donors appeared upon short-term cell exposure presumably under non-equilibrium conditions, complicating the comparison of cell responses. Nevertheless, these results suggest that in comparison with NaHS, the dithiophosphates donate H_2_S at a lower concentration as if some non-hydrolyzed molecules delivered and acted intracellularly. The existing reports provide quite variable data on donor-mediated change in cellular H_2_S depending on the properties of donors and fluorescent probes and the assay conditions [55,56,57,58,59,60].

Considering the redox activity of H_2_S and glutathione, cellular effects of the dithiophosphates were additionally assessed according to changes in intracellular levels of ROS and GSH. For this purpose, C2C12 myoblasts and myocytes were used as a useful in vitro model of skeletal and cardiac muscles under physiological and pathological conditions [61,62,63,64,65]. These cells were used to study skeletal muscle atrophy due to apoptosis, oxidative stress and inflammation and to assess myoprotective molecules [66,67]. The cytoprotective activity of NaHS was recently shown in homocysteine-treated C2C12 myoblasts and untreated C2C12 myotubes, where released H_2_S, respectively, reversed oxidative and endoplasmic reticulum stresses [68] and caused the upregulation of antioxidant-related genes, also decreasing ROS and homocysteine levels [69].

The differentiation of C2C12 cells was accompanied by a considerable ROS overproduction as well as a moderate and probably compensatory increase in GSH levels in accordance with earlier observations [70,71]. Exogenic GSH at excessive concentrations was also used to probe the cell redox state via changes in MCB fluorescence [40]. The detected decrease in the MCB signal of GSH-treated cells (Figure 4) suggests a saturated GSH pool in intact myoblasts and myocytes, meaning that its excessive elevation could provoke reductive stress [72,73,74].

The effects of dithiophosphates and NaHS were assessed in different concentration ranges, i.e., 0.1–10 μM and 0.1–1 mM, respectively, given the noticeable difference in their activities. The myocytes exposed to GSH–DTP, GSSG–(DTP)_2_ and NaHS showed a decreased MCB signal (Figure 4B), presumably due to redox imbalancing of the cells, similarly to that induced by exogenic GSH. The effect was observed only at upper concentrations of NaHS and weakly depended on the concentration of the glutathione derivatives. DTP did not significantly decrease the MCB signal in myocytes (Figure 4B). Furthermore, the donors affected the ROS level in myocytes differently, decreasing it in the case of GSSG–(DTP)_2_, DTP, GSH and NaHS and modulating it in the case of GSH–DTP (Figure 5B).

The results suggest that the compounds are potentially capable of inhibiting overproduced ROS in myocytes, which can be accompanied by a decrease in GSH content. GSSG–(DTP)_2_ and especially GSH–DTP tend to cause more complicated responses attributed to enhanced redox-modulating activity. These data are in accordance with concentration-dependent anti- and prooxidant properties of H_2_S in vitro and in vivo [75,76,77].

Considering the regulatory role of H_2_S in myocardial contractile activity [18,78,79,80], the inotropic function of the rat atrial myocardium in the presence of synthesized donors was evaluated in comparison with NaHS with established activity.

Previously, the dose-dependent inhibition of myocardium relaxation was revealed in isolated Langendorff hearts of frogs and rats treated with NaHS [19,20,81]. Furthermore, NaHS and L-cysteine decreased the contractility of isolated mouse atrium, while it was partially restored by glibenclamide [42], the inhibitor of K(ATP) channels. In single cardiomyocytes isolated from rat ventricles, NaHS reduced Ca^2+^-transients and contractions as well as the action potential amplitude [17,21].

Similar to NaHS, the dithiophosphates ensured a negative inotropic effect, decreasing the maximum velocity of the contraction and relaxation. However, the effect of the latter donors progressed more slowly and steadily, reaching a higher inhibition of the contractile force in the case of GSH–DTP and DTP (Figure 6 and Figure S3). This supports a more sustained release of H_2_S by the dithiophosphates in myocardium tissues compared to NaHS, which generates H_2_S in several seconds as a result of the dissociation and interaction of HS^−^ ions formed with H^+^ [82]. Depending on the pH, temperature and salinity, only 11–13% of HS^−^ are converted to H_2_S, and up to 50% of H_2_S is evaporated within 3 minutes [4,83,84]. The negative lusitropic effects of H_2_S are probably mediated by the S-sulfhydration of phospholamban, a key regulator of contraction and relaxation via Ca^2+^ uptake in the sarcoplasmic reticulum [19,83].

Altogether, these data suggest that the dithiophosphates are effective H_2_S donors with more sustained effect on atrial contraction compared to NaHS. Among them, GSH–DTP showed both the highest and most prolonged activity in accordance with its higher H_2_S donation potential (Figure 2A). The inferior activity of GSSG–(DTP)_2_ compared to both GSH–DTP and DTP could presumably be attributed to its lower availability at the tissue level or other factors associated with in situ redox conversions of released H_2_S and the glutathione component. Clarification of the specific activities of GSH–DTP and GSSG–(DTP)_2_ will be performed elsewhere.

## 4. Materials and Methods

### 4.1. Materials

Reduced glutathione (purity 98%) and methanol was purchased from Acros Organ-ics (Geel, Belgium). Monochlorobimane (MCB) dye was purchased from ThermoFisher Scientific (Waltham, MA, USA). 7-azido-4-methylcoumarin (AzMC), sodium hydrosulfide (NaHS), 2′,7′-dichlorofluorescin diacetate (DCFDA) and 3-(4,5-dimethyl-thiazol-2-yl)-2,5-diphenyltetrazolium bromide (MTT) were purchased from Sigma-Aldrich. Milli-Q grade water (Milli-Q^®^ Advantage A10, Merck Millipore, St. Louis, MO, USA) was used to prepare buffers and solutions. Cell culture media and reagents were purchased from Paneco (Moscow, Russia) and Sigma-Aldrich (St. Louis, MO, USA).

**Figure 7:**
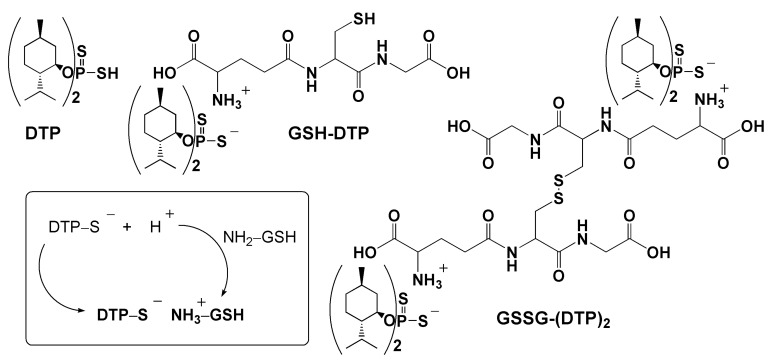
Structural formulas of O,O–(–)–dimenthyl dithiophosphoric acid (DTP) and its salts with reduced and oxidized glutathione denoted as GSH–DTP and GSSG–(DTP)_2_. Frame: scheme for GSH–DTP formation.

Ammonium salts of glutathione and O,O-(–)-dimenthyl dithiophosphoric acid (Figure 7) were synthesized and characterized as detailed earlier [39]. Stock solutions of the compounds were prepared in methanol.

### 4.2. Cell Culture and Viability Assessment

C2C12 immortalized mouse myoblast cell line (Institute of Cytology Russian Academy of Science) was used. The cells were cultured aseptically in DMEM containing 10% fetal bovine serum (FBS), 4.5 g/L glucose, 2 mM L-glutamine, 100 U/mL penicillin and 100 μg/mL streptomycin at 37 °C in humidified air atmosphere with 5% CO_2_.

Cell viability was assessed using an MTT proliferation assay [85] upon culturing in the presence of H_2_S donors in a concentration range of 0.05–100 μM for 72 h. Cell viability was presented in percent relative to untreated cells (100% viability). Half-maximal inhibitory concentrations (IC_50_) were calculated from concentration–viability relationships using GraphPad Prizm 5 software. The data were presented as mean ± SD (*n* = 3).

For cell differentiation into myocytes, the medium was replaced by DMEM containing 2% horse serum, 1 g/L glucose, 1% L-glutamine, 0.4 μg/mL dexamethasone, 100 U/mL penicillin and 100 μg/mL streptomycin [86]. Over the course of 5 days, the cells were morphologically differentiated and fused to form myotubes.

### 4.3. Detection of H_2_S Release

The kinetics of H_2_S release by the glutathione dithiophosphates was studied in PBS (pH = 7.4) using a 7-azido-4-methylcoumarin (AzMC) probe. H_2_S donors (1 mM) were dissolved in PBS, mixed AzMC probe (10 μM) and incubated at room temperature for 4 h. The emission spectra of AzMC were recorded on an FL3-221-NIR spectrofluorometer (Horiba Jobin Yvon, Kyoto, Japan) using a 1 cm quartz cuvette at λ_ex_ = 365 nm. The fluorescence intensity of AzMC in the presence of compounds was measured every 5 min. 

### 4.4. Laser Scanning Confocal Microscopy (LSCM)

C2C12 myoblasts were grown overnight on coverslips in a 6-well plate at a density of 50 × 10^3^ cells per coverslip. LSCM analysis of the cells was performed in an Attofluor™ cell chamber (ThermoFisher Scientific, Waltham, MA, USA) and using an LSM 780 confocal microscope (Carl Zeiss, Jena, Germany) with a 405 nm laser. The cells were stained with 10 µM AzMC in Hank’s balanced salt solution (HBSS) for 30 min followed by treatment with 10 µM dithiophosphates or 100 µM NaHS for 20 min. The relative fluorescence of the cells in LSCM images was calculated using NIH ImageJ 1.48v software and presented as mean ± SD (*n* = 12). Statistical significance was determined via one-way analysis of variance (ANOVA) followed by Tukey’s Multiple Comparison post-test (* *p* < 0.05, ** *p* < 0.01, *** *p* < 0.001).

### 4.5. GSH and ROS Detection

The effects of compounds on cytoplasmic ROS and reduced glutathione (GSH) levels were analyzed with DCFDA and MCB probes, respectively, as detailed previously [40,85]. The cells were seeded in a 96-well plate at a density of 20 × 10^3^ cells per well in the culture medium and were grown overnight. The cells were exposed to GSH (1, 10 mM), NaHS (0.1, 1 mM) or dithiophosphates (0.1–1 μM) for 1 h in HBSS, followed by staining with 5 µM MCB for 1 h or 20 μM DCFDA for 20 min in CO_2_-incubator. Cell fluorescence intensity was registered at λ_ex/em_ = 380/480 nm (MCB) and λ_ex/em_ = 490/526 nm (DCFDA) on an Infinite M200 PRO microplate analyzer (Tecan, Männedorf, Switzerland). Relative GSH and ROS levels were presented in percent versus untreated cells (100%) as mean ± SD. Statistical significance was determined using two-way analysis of variance (ANOVA) followed by a Bonferroni post-test to compare replicate means by row (* *p* < 0.05, ** *p* < 0.01, *** *p* < 0.001).

### 4.6. Study of Isolated Rat Right Atrial Contraction

Animal experiments were performed using Wistar rats (250–300 g) in accordance with the EU Directive 2010/63/EU and approved by the Local Ethical Committee of Kazan Federal University (protocol No 33 from 25 November 2021). The rats were euthanized under deep isoflurane anesthesia, and the right atria were dissected from the heart. Atrial strips of 4–6 mm in length and 2–3 mm in thickness were vertically mounted in a 20 mL chamber with Tyrode’s solution composed of (mM): NaCl = 130, KCl = 5.6, NaH_2_PO_4_ = 0.6, MgCl_2_ = 1.1, CaCl_2_ = 1.8, NaHCO_3_ = 20 and glucose = 11, pH = 7.4. The solution was bubbled with carbogen (95% O_2_, 5% CO_2_). The atrial stripes were stimulated via two platinum electrodes at a frequency of 0.1 Hz. Muscle contraction was recorded using a transducer TSD 125C (Biopac Systems Inc., Goleta, CA, USA) and analyzed using a computerized recording system as described previously [41,87,88].

Following equilibration for 30–60 min to stabilize atrial contraction, H_2_S donors were applied at a concentration of 200 μM, and the measurements were repeated. The registered responses were normalized relative to the control contraction. The lack of effect of solvent used (0.001% methanol in Tyrode’s solution) was confirmed. The amplitude of contraction, maximal velocity of contraction (MVC) and maximal velocity of relaxation (MVR) were analyzed. Data are expressed as mean ± S.E.M (*n* = 6–8). To test the normal distribution of the data, the Fisher F-test and Shapiro–Wilk test were applied using OriginPro 8.5 software. To compare two independent groups and paired data, the Mann–Whitney U test and the Wilcoxon matched pairs test were used, respectively.

## Data Availability

The data presented in this study are contained within the article and Supplementary Materials.

## Supplementary Materials

The supporting information can be downloaded at: https://www.mdpi.com/article/10.3390/ijms241311063/s1.
