# Supplementary material for: Characterization of Glutathione Dithiophosphates as Long-Acting H2S Donors
Source: Int J Mol Sci. 2023 Jul 4;24(13):11063. doi: 10.3390/ijms241311063 (PMC10342186; doi:10.3390/ijms241311063)
Supplement: Supplementary file 1 [file ijms-24-11063-s001.zip › ijms-2471603-supplementary.pdf]

Characterization of glutathione dithiophosphates as long-acting H_2S donors

Rezeda A. Ishkaeva [1,2], Nail N. Khaertdinov [1], Aleksey V. Yakovlev [1], Marina V. Esmeteva [1,2], Diana V. Salakhieva [1,2], Ilyas S. Nizamov [3,4], Guzel F. Sitdikova [1,*], and Timur I. Abdullin [1,2]

[1] Institute of Fundamental Medicine and Biology, Kazan (Volga Region) Federal University, 18 Kremlyovskaya St., 420008 Kazan, Russia
[2] Scientific and Educational Center of Pharmaceutics, Kazan (Volga Region) Federal University, 18 Kremlyovskaya St., 420008 Kazan, Russia
[3] Alexander Butlerov Institute of Chemistry, Kazan (Volga Region) Federal University, 420008 Kazan, Russia
[4] Arbuzov Institute of Organic and Physical Chemistry, FRC Kazan Scientific Center of RAS, 8 Arbuzov St., 420088 Kazan, Russia
* Correspondence: guzel.sitdikova@kpfu.ru; Tel.: +7-(843)-206-52-34

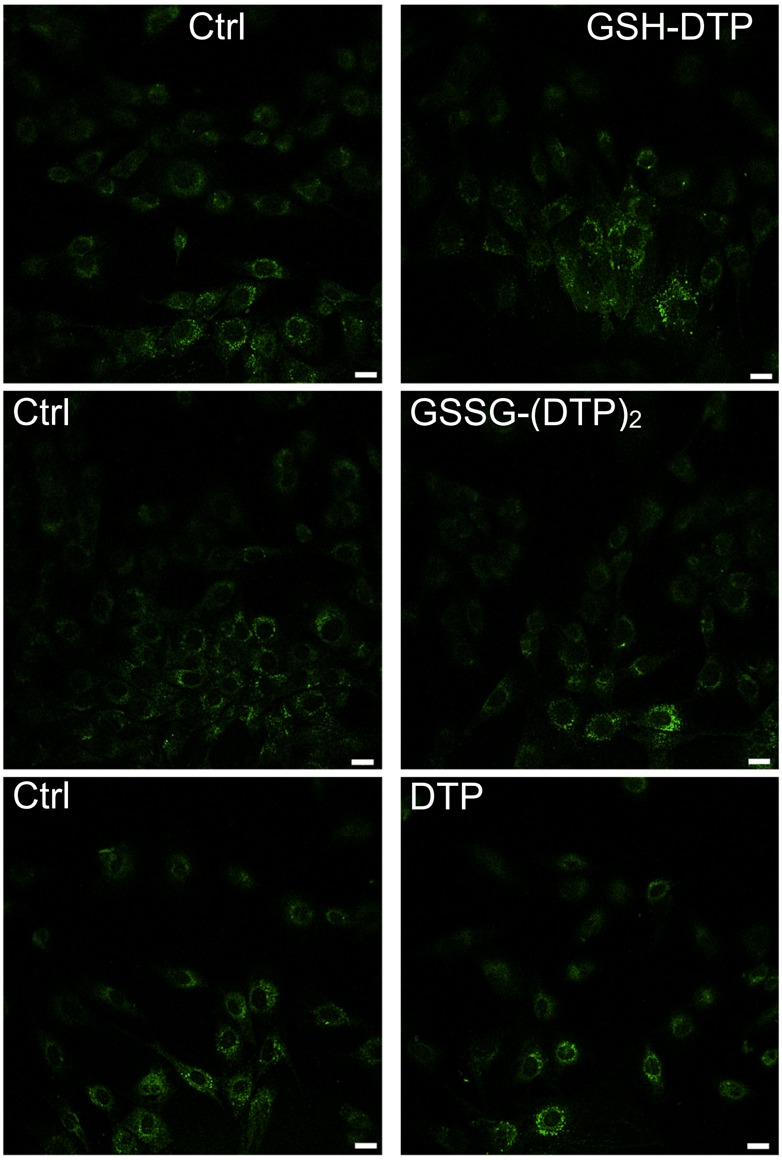

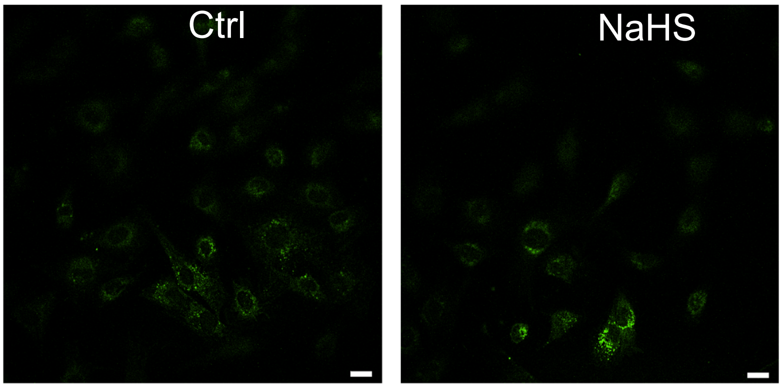

Figure S1. Effect of glutathione dithiophosphates, DTP and NaHS on H_2S level in C2C12 mioblasts according to AzMC fluorescence. Representative LSCM images of control and GSH–DTP-treated cells (scale bar = 10 μm). The pre-stained cells were exposed to the compounds for 20 min.

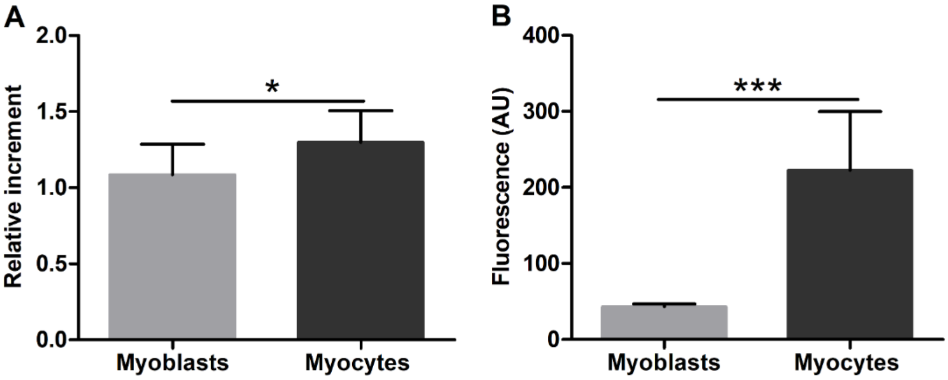

Figure S2. Relative GSH (**A**) and ROS (**B**) content in C2C12 myoblasts and C2C12 myocytes according MCB microplate assay [Ishkaeva, *et al.*, 2022] and DCFDA fluorescence. Mean ± SD are shown ($^{*}p < 0.05$, $^{***}p < 0.001$).

Citation: Ishkaeva, R.A.; Khaertdinov, N.N.; Yakovlev, A.V.; Esmeteva, M.V.; Salakhieva, D.V.; Nizamov, I.S.; Sitdikova, G.F.; Abdullin, T.I. Characterization of Glutathione Dithiophosphates as Long-Acting H_2S Donors. *Int. J. Mol. Sci.* **2023**, *24*, 11063. https://doi.org/10.3390/ijms241311063

Academic Editor: Sonia Melino

Received: 10 June 2023
Revised: 28 June 2023
Accepted: 30 June 2023
Published: 4 July 2023

Copyright: © 2023 by the authors. Submitted for possible open access publication under the terms and conditions of the Creative Commons Attribution (CC BY) license (https://creativecommons.org/licenses/by/4.0/).

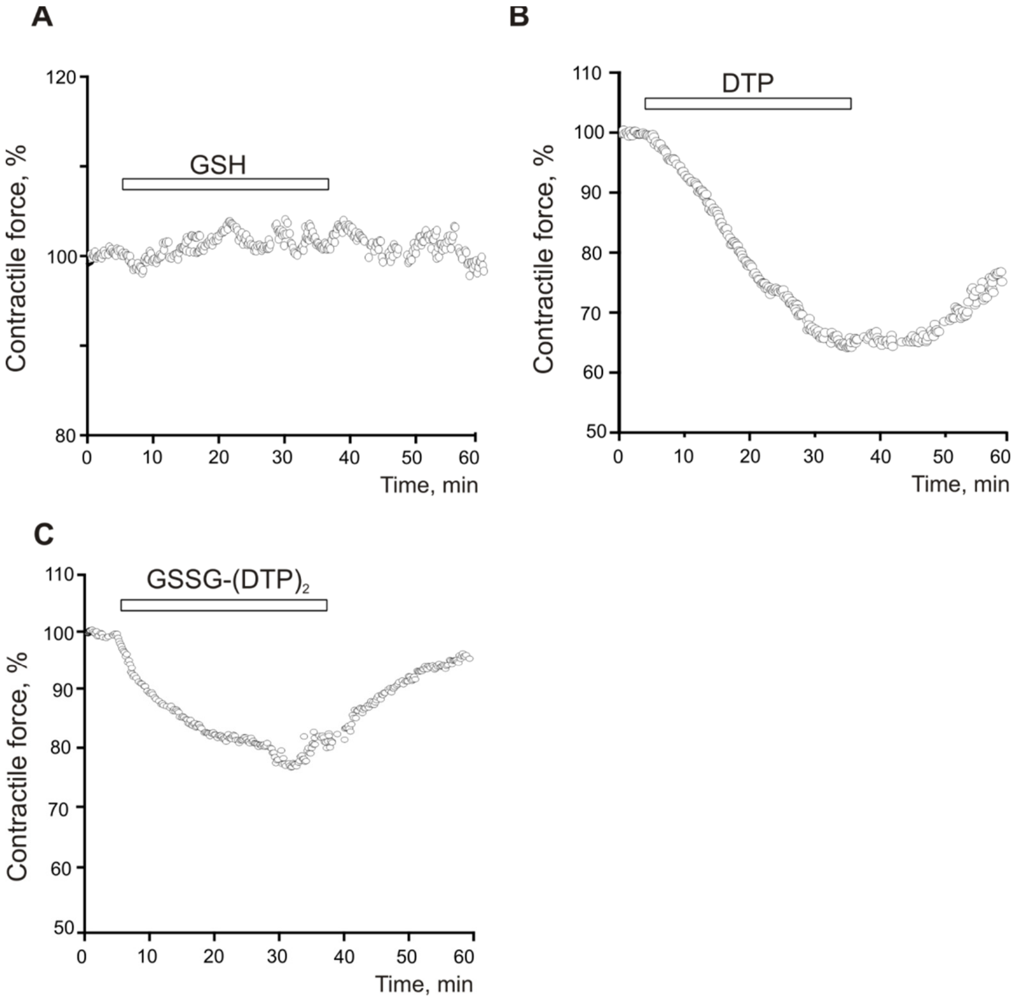

Figure S3. Effects of H_2S donors on the contractile force of rat atrium. The average time courses of negative inotropic action of 200 μM GSH (**A**), 200 μM DTP (**B**) and GSSG-$(DTP)_2$ (**C**) The time of application is indicated by the bar.
